# Improving the Fracture Toughness of Boron Carbide via Minor Additions of SiC and TiB_2_ Through Hot-Press Sintering

**DOI:** 10.3390/ma17246233

**Published:** 2024-12-20

**Authors:** Juhan Ka, Kyoung Hun Kim, Woohyuk Choi, Sungmo Jung, Tae Hwan Lee, Hyun Sik Kim, Heesoo Lee, Jae Hwa Lee

**Affiliations:** 1 Analysis & Standards Center, Korea Institute of Ceramic Engineering & Technology (KICET), 101 Soho-ro, Jinju-si 52851, Republic of Korea; kajh317@kicet.re.kr (J.K.); khkim@kicet.re.kr (K.H.K.); hyunkim@kicet.re.kr (H.S.K.); 2 School of Materials Science & Engineering, Pusan National University, 2 Busandaehak-ro 63beon-gil, Busan 46241, Republic of Korea; 3 Material R&D Center, Samyang Comtech Co., Ltd., 81 Mansam-ro, Gwangju-si 12726, Republic of Korea; goomn@samyangct.com (W.C.); jsm0409@samyangct.com (S.J.); hwan88@samyangct.com (T.H.L.); 4 Department of Materials Science & Engineering, Myongji University, 116 Myongji-ro, Yongin-si 17058, Republic of Korea

**Keywords:** B_4_C composites, hot pressing, microstructure, fracture mode, toughness mechanism

## Abstract

Boron carbide (B_4_C) is an essential material in various high-performance applications due to its light weight and hardness. In this work, B_4_C-based composites were fabricated via a powder route consisting of powder mixing, precursor preparation, and hot-pressing under vacuum. The composites’ mechanical properties and microstructure were analyzed to investigate the effect of adding minor second-phase particles. In addition to homogenizing the grain size, the addition of SiC (≤10 wt%) to B_4_C increased its strength and improved its fracture toughness, with values reaching 551 MPa and 3.22 MPa m^1/2^, respectively. Meanwhile, the addition of TiB_2_ (≤10 wt%) significantly improved the strength and fracture toughness only, with values reaching 548 MPa and 3.92 MPa m^1/2^, respectively, with only a minimal decrease in hardness. Microstructural analysis revealed that the second-phase particles were uniformly distributed and reduced the average grain size, contributing to the increase in strength. Additionally, the TiB_2_ particles impeded crack propagation and induced crack deflection at the interface, indicating the formation of an intergranular fracture mode. On the contrary, the addition of SiC primarily resulted in transgranular fracture behavior, though it still improved the toughness of the B_4_C. These results suggest that small amounts of SiC and TiB_2_ can effectively enhance the mechanical properties of B_4_C ceramics while maintaining the lightweight characteristics critical for military and aerospace applications.

## 1. Introduction

The aerospace, defense, and nuclear power generation industries have emerged as focal points of future industrial development; as such, the demand for materials that can withstand extreme conditions, such as ultra-high temperatures, high pressures, oxidation, and corrosion, is rising. Boron carbide (B_4_C) is a material with a series of excellent mechanical and chemical properties, including extremely high hardness (>30 GPa), low density (2.52 g/cm^3^), a high melting point (2350 °C), and high abrasion resistance [1,2,3]. These characteristics are due to the high fraction of covalent bonding in its structure [4]. Based on the Vickers hardness at room temperature, B_4_C is the third-hardest natural material after diamond and cubic boron nitride [5]. Moreover, the density of B_4_C is lower than that of most structural ceramics, making it an excellent lightweight armor material with extremely high hardness that is popular in the construction of military helicopters [6,7]. Compared to oxide-based ceramics such as alumina and zirconia, B_4_C offers superior hardness and a significantly higher strength-to-weight ratio. These characteristics make B_4_C particularly advantageous for military and aerospace applications, in which reducing weight without compromising performance is critical. With its potential to revolutionize the above-mentioned industries, it is crucial to address the current limitations of B_4_C, such as its low fracture toughness (only about 2.2 MPa m^1/2^), which hinders its extensive industrial application [8,9,10].

Sintering B_4_C to high densities inevitably requires the use of additives or high external pressures, which contribute to its low self-diffusion coefficient, high grain boundary slip resistance, and low plasticity at high temperatures [2,11,12]. While the method of pressureless sintering only densifies pure B_4_C ceramics to less than 80% of their theoretical density even at temperatures above 2300 °C, in combination with abnormal grain growth, hot-press sintering can guarantee full densification approaching the theoretical density (2.52 g/cm^3^) and a significant reduction in the sintering temperature to 2000 °C [13,14]. Also, hot-press sintering ensures high densification efficiency and uniform microstructures at relatively lower sintering temperatures, though it requires longer processing times and higher energy consumption. Sintering aids are often added to improve the low sinterability of B_4_C by lowering the surface energy of grain boundaries and promoting material spreading during sintering, thereby enabling the fabrication of a compact B_4_C body [15]. Among various types of sintering aids, non-oxides like carbide and boride (e.g., SiC, TiC, TiB_2_, ZrB_2_, CrB_2_, and WC) have been most often employed in recent decades because they not only facilitate the densification of B_4_C-based ceramics with lower sintering temperatures but also induce the formation of a secondary phase in the microstructure that can impart improved strength and toughness to the materials [10,16,17,18,19,20,21].

Previous studies consistently identified SiC and TiB_2_ as the most suitable second phases for addition to B_4_C ceramics based on their stiff yet light properties [4,11,22,23,24,25]. These second-phase particles play a crucial role in refining the grain structure and enhancing the roughness and strength of B_4_C ceramics, serving as reinforcement phases. For instance, So et al. reported B_4_C-SiC composites fabricated via hot-press sintering at 2000 °C with 40 MPa of pressure in which the bending strength and Vickers hardness at SiC 50 wt% were 645 MPa and 30.6 GPa, respectively [11]. Similarly, adding TiB_2_ to B_4_C significantly improved its mechanical properties, particularly its fracture toughness [17,19,26]. Yamada et al. synthesized a B_4_C-TiB_2_ composite (20 mol% TiB_2_) with both a high strength of 866 MPa and an improved fracture toughness of 3.2 MPa·m^1/2^ using fine B_4_C powders containing 0.3–0.5 wt% of aluminum and iron impurities [26]. The toughening effect in the B_4_C-TiB_2_ composites can also be attributed to the thermal expansion mismatch between the matrix and dispersed particles, which causes residual stresses. Likewise, the addition of SiC and TiB_2_ particles in the B_4_C matrix is considered to be the simplest yet most effective means of improving its sintering behavior and mechanical properties simultaneously. However, increasing toughness through ceramic matrix composites often requires the use of large amounts of heavier additives (e.g., the densities of SiC and TiB_2_ are 3.21 and 4.52 g/cm^3^, respectively), compromising the low density of B_4_C, a property essential for its military and aerospace applications. It is worth noting that the effects of SiC and TiB_2_ on the toughening of B_4_C need to be investigated more in terms of crack impeding and deflection.

Building on the previous findings, this study introduces a novel approach by limiting the amount of SiC and TiB_2_ additives to ≤10 wt%, maintaining the low density of B_4_C while enhancing its fracture toughness and flexural strength. Furthermore, microstructural analysis provides new insights into the distinct roles of SiC and TiB_2_ in crack behavior and toughening mechanisms. In this regard, we fabricated B_4_C-based composites with small amounts of SiC and TiB_2_ dispersed in the B_4_C matrix (referred to as B_4_C-*X*-*Y*%, where *X* and *Y* represent the type and amount of additive, respectively), utilizing hot-press sintering without other sintering aids. The as-synthesized composites showed a significant increase in fracture toughness and flexural strength but no dramatic changes in density, elastic modulus, or Vickers hardness. Furthermore, microstructural analysis using scanning electron microscopy (SEM) revealed that the combination of the crack-impeding effect of TiB_2_ particles and crack deflection at the interface resulted in an intergranular fracture mode. On the contrary, the addition of SiC did not necessarily result in an intergranular fracture mode but rather transgranular behavior while enhancing the toughness of the B_4_C.

## 2. Materials and Methods

### 2.1. Materials and Synthesis

The powders used in the present study are commercially available and were used as raw materials: B_4_C (HS Grade, Höganäs AB, Höganäs, Sweden, d_50_ = 3 μm), SiC (Sintex 15C, Kymera International, Oslo, Norway, d_50_ = 0.1 μm), and TiB_2_ (Grade T05, Zibo Jonye Ceramic Technologies Co., Ltd., Zibo, China, d_50_ = 8 μm). To mix and homogenize the powders, mechanical milling was performed with SiC balls (φ = 10 mm) in a laboratory Nalgene bottle, using ethanol as a solvent for 2 h. The milling speed was 185 rpm, the solvent-to-powder weight ratio was 7:10, and the ball-to-power volume ratio was 1:1. Details of the weight ratios for SiC or TiB_2_ to B_4_C in the prepared mixtures are provided in Table S1. The as-obtained slurry was placed on a hot plate and heated overnight to evaporate the ethanol while stirring vigorously to prevent separation. Then, the mixed powders were completely dried at 100 °C for 24 h in a drying furnace and sieved through a 200-mesh sieve to minimize powder agglomeration for further use. The prepared powder mixture was poured into a coin-shaped graphite mole (φ = 60 mm) and heated in a vacuum, first to 500 °C for 1 h and then to 2000 °C, where it was held for another 1 h under a pressure of 40 MPa, using a uniaxial pressurized sintering unit (SHP-200-30T, Samyang Ceratech Co., Ltd., Incheon, Republic of Korea). After sintering, the sample was naturally cooled to room temperature (Figure S1). Notably, to prepare an intrinsic B_4_C sample, the raw B_4_C powder was directly sintered without additional mixing and drying procedures. Coin-shaped composite samples with a diameter of 60 mm and a height of 15 mm were fabricated for each mixture. These samples were machined into specific specimens for various analyses. For each composite, specimens were prepared for evaluation as follows: 10 specimens for flexural strength testing, 2 for hardness measurements, and 5 for density evaluation. All tests were conducted according to the methods described to ensure statistical reliability and consistency. This comprehensive approach enabled a detailed evaluation of the mechanical and microstructural properties of the composites.

### 2.2. Characterization

The density of the sintered body was measured using Archimedes’ method, using distilled water as the immersion medium. X-ray diffraction analysis (XRD; SmartLab, Rigaku, Tokyo, Japan) was performed with Cu K_α_ (λ = 1.540598 Å) radiation at 45 kV and 200 mA. Vickers indentation tests were carried out using a hardness tester (HM-200, Mitutoyo, Kanagawa, Japan) with an indentation load of 9.8 N and a dwell time of 15 s. The fracture toughness (K_IC_) was calculated from the length of the radial cracks of each indent using the equation K_IC_ = 0.018 × (E/H)^0.5^ × (Pc^−1.5^), where E is the Young’s modulus in GPa, H is the Vickers hardness in GPa, P is the indentation load (9.8 N), and c is the average crack length (the length from the center of the indent to the crack tip in meters) [7,22,27]. It should be noted that before the Vickers hardness and fracture toughness measurements, the surfaces of the sintered specimens were first ground using SiC grinding papers and then mirror-polished with a diamond suspension. The flexural strength was measured using a three-point bending method on a universal testing machine (RB-301 unitech M, R&B Inc., Daejeon, Republic of Korea), where the dimensions of the testing bars were 3 mm × 4 mm × 37 mm. The obtained mechanical values were the mean and standard deviation from 10 bars for flexural strength and at least 5 indentations for hardness and fracture toughness, respectively. Additionally, the elastic modulus was measured using a pulse–echo method with an elastic modulus tester (TDS-220, Tektronix, Beaverton, OR, USA). Microstructural investigations of the composites were conducted via SEM (JSM-7610F, JEOL Ltd., Tokyo, Japan) with an energy-dispersive spectrometer (EDS) analysis system, before which each mirror-polished specimen was electrolytically, etc., ed in 1% KOH solution at 5 V for up to 1 min.

## 3. Results and Discussion

### 3.1. Effect of SiC Content on Microstructure and Mechanical Properties of B_4_C Matrix Composite

**Figure 1:**
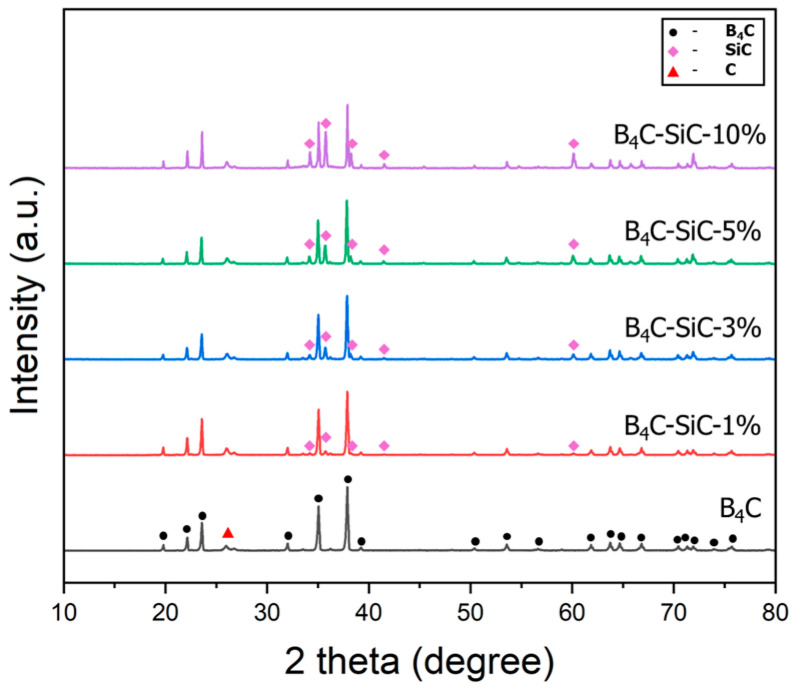
XRD patterns of B_4_C-SiC composites with varying SiC contents (1%, 3%, 5%, and 10%).

**Figure 2:**
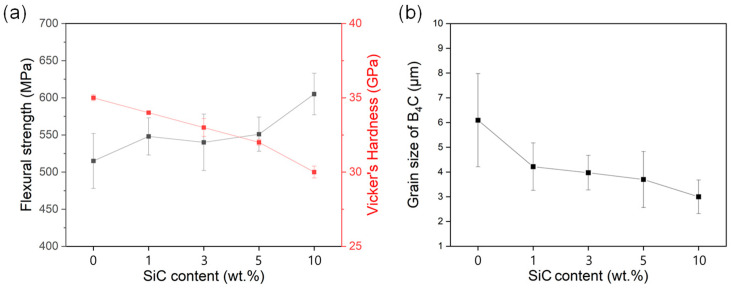
Mechanical properties of B_4_C-SiC composites with varying SiC contents (1%, 3%, 5%, and 10%): (**a**) flexural strength and Vickers hardness, and (**b**) grain size of B_4_C.

**Figure 3:**
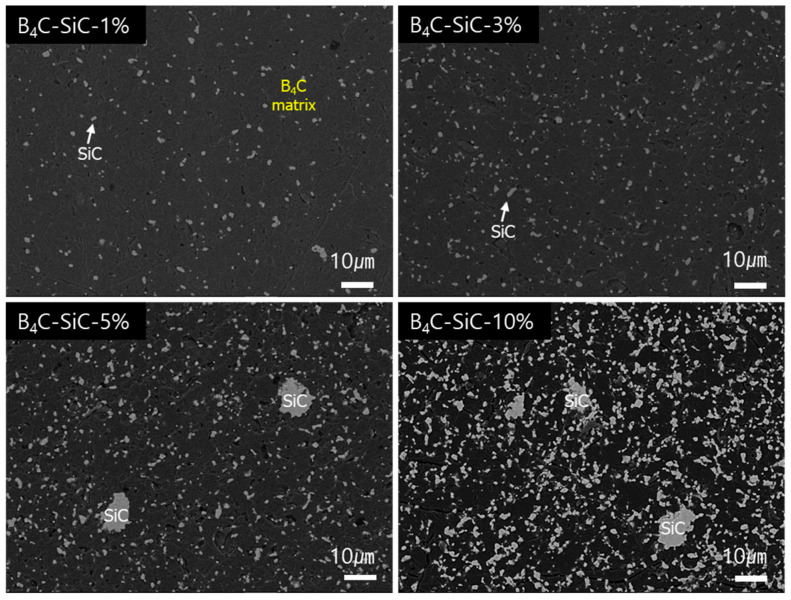
SEM images of B_4_C-SiC composites with varying SiC contents (1%, 3%, 5%, and 10%), captured in BSE mode.

The effect of the SiC content on the phase compositions, physical properties, and microstructures of the B_4_C-SiC ceramic composites prepared through hot-press sintering at 2000 °C is shown in Figure 1, Figure 2 and Figure 3. Figure 1 shows that the XRD patterns of the composites only indicate the peaks of B_4_C and SiC, and no other phases are detected. As the SiC content increased from 1 to 10 wt%, the XRD peaks of the SiC phase became more distinct. Additionally, the XRD results show no peak shift for the B_4_C and SiC phases, suggesting no phase transitions or changes in the crystal structure. Comprehensively, no reactions occur between the SiC and B_4_C phases during the hot-pressing period, at least not enough to be detected by XRD (approximately less than 1%), and the SiC particles stably exist as an independent phase within the B_4_C matrix. Thus, following our design, it was confirmed that SiC is a feasible additive for fabricating B_4_C-based composites.

Table 1 contains the bulk density and relative density results of B_4_C-SiC ceramics with different SiC contents. The density decreases with the increase in SiC content because the density of SiC (3.21 g/cm^3^) is higher than that of B_4_C (2.51 g/cm^3^). Nevertheless, a relative density of over 99% is achieved for the pure B_4_C and all the B_4_C-SiC ceramic composites owing to the hot-pressing processes, which offer a uniform distribution of heating and pressure to facilitate the production of fully dense samples with consistent microstructural properties (Table S2) [11,25,26,27,28,29]. Variations in the physical properties of the B_4_C-SiC ceramics are also listed in Table 1. First, the Vickers hardness of the composites shows a decreasing trend with the increase in SiC content, dropping from 34.8 to 29.9 GPa when the amount of additional SiC reaches 10 wt% (Figure 2a). Intrinsically, SiC has a lower hardness (25–30 GPa) than B_4_C (>30 GPa), and the hardness of ceramic composites is determined by the nature of their composition. Thus, it is natural that the overall hardness of the B_4_C-SiC composites decreases gradually with an increase in SiC content as the composites had reached maximum density and cannot change much in hardness. Notably, it has been observed that the hardness of B_4_C-based composites decreases more drastically when SiC is added in proportions greater than 20 wt% [4]; however, the B_4_C-SiC-10% still shows a high hardness value of above 30 GPa.

On the other hand, the flexural strength curve in Figure 2a shows a tendency to increase as the amount of SiC increases to 10 wt%. As shown in Figure 2b, the grain size of the B_4_C decreases as the amount of SiC increases. In other words, SiC inhibited grain growth, resulting in a denser microstructure (Figure S2). According to the Hall–Petch effect, this phenomenon indicates that as the grain size decreases, the number of grain boundaries increases, suppressing crack propagation [25,30]. As a result, the flexural strength of the composite increased, with the highest flexural strength (605 MPa) achieved when 10 wt% of SiC was added (Table 1 and Figure 2a). The pinning effects of the fine SiC particles (d_50_ = 0.1 μm) limit grain growth, which improves the overall physical properties of the B_4_C-SiC ceramics when considering that the composites still maintained high hardness levels over 30 GPa.

The SEM images captured in BSE mode (backscattered electron mode) using the contrast caused by differences in the atomic numbers of the elements in the sample allow for the phases to be clearly distinguished. The contrast between SiC and B_4_C was evident, allowing us to observe how SiC was distributed within the composite. SiC was uniformly distributed at 1 to 3 wt%, but agglomeration was partially observed starting with the 5 wt% addition, as shown in Figure 3. Despite the presence of minor agglomeration regions, especially at higher SiC contents such as 5 wt% and 10 wt%, the SEM images at higher magnification (Figure S3) still prove significant grain refinement and fragmentation of SiC particles through all the composites. Generally, when an excessive amount of secondary phase is added, agglomeration occurs, which negatively affects the microstructure of the composite and may lead to a decrease in strength [5,23,26]. However, since the amount of SiC added in this study was small (≤10 wt%), the number of agglomerations was low, and its impact was limited. In other words, even though some agglomeration occurred, the small amount did not significantly affect the uniformity of the microstructure, and the grain-growth-inhibiting effect of SiC still contributed to preventing a reduction in strength. These results suggest that the composite’s performance can be optimized with small amounts of additives and that the mechanical performance of the composite can be improved while minimizing the adverse effects of agglomeration.

### 3.2. Microstructure and Mechanical Properties of B_4_C-TiB_2_ Composites

**Figure 4:**
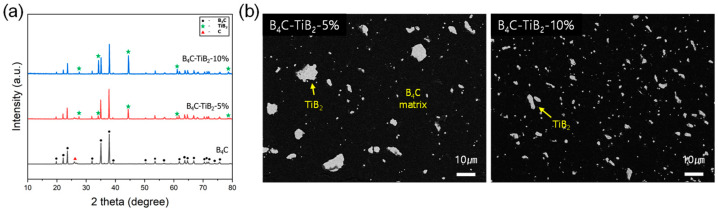
(**a**) XRD patterns and (**b**) SEM image in BSE mode of the B_4_C-TiB_2_-5% and B_4_C-TiB_2_-10% composites.

Like SiC, TiB_2_ has been widely used as a secondary-phase reinforcement material for B_4_C due to its high melting point (3230 °C) and hardness [7,22,26]. As shown in Figure 4a, the XRD analysis results indicate that the crystal structure changes with the addition of TiB_2_, showing a similar trend to that of SiC. Compared to pure B_4_C, the characteristic diffraction peaks of TiB_2_ appeared clearly in the composites containing 5 wt% and 10 wt% TiB_2_, indicating that TiB_2_ exists stably as an independent phase within the composites. As the amount of TiB_2_ increased, the intensity of the XRD peaks gradually increased, showing the same pattern as with the addition of SiC. TiB_2_ and SiC formed more distinct crystal structures within the composite as their content increased. This result demonstrates that TiB_2_ and SiC remain independent phases without causing phase transitions or chemical changes with B_4_C to be detectable by XRD, even after sintering. In conclusion, the XRD analysis results show that TiB_2_ exhibits a similar crystal structure and distribution pattern to SiC, with both substances existing independently and stably within the composite. TiB_2_ and SiC each play an essential role in enhancing the physical performance of the composite, and their stability was confirmed in the XRD patterns.

The distribution of TiB_2_ particles within the composites containing 5 wt% and 10 wt% TiB_2_ can be seen in Figure 4b. Although some agglomeration was observed at 5 wt% TiB_2_, the particles were more uniformly distributed at 10 wt%. This indicates that dispersibility improved as the TiB_2_ content increased, forming a uniform microstructure in the 10 wt% TiB_2_ composite. Like SiC, TiB_2_ inhibited grain growth within the composite and reduced grain size, improving the composite’s mechanical strength (Figure S4). It is particularly noteworthy that, unlike SiC, TiB_2_ did not cause a reduction in hardness. Even with the addition of 5 wt% and 10 wt% TiB_2_, the hardness remained around 35 GPa, indicating that the addition of TiB_2_ did not negatively affect the hardness of the composite. This demonstrates that the minor addition of TiB_2_ contributed to improving the composite’s strength by forming a uniform microstructure and inhibiting grain growth while maintaining mechanical performance and without a decrease in hardness. Notably, it was proven that even a small addition of TiB_2_ (≤10 wt%) enhanced the overall performance of the B_4_C composites, providing important insights into optimizing their performance.

### 3.3. Reinforcement Mechanism of B_4_C Composites with Small Additions of SiC and TiB_2_

**Figure 5:**
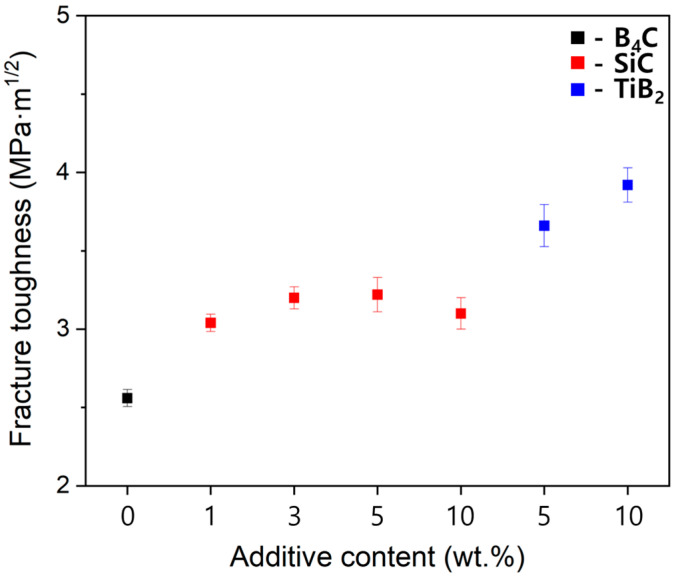
Fracture toughness of B_4_C composites as a function of SiC and TiB_2_ contents.

As shown in Figure 5, the addition of small amounts of SiC and TiB_2_ to B_4_C significantly increases the composite’s fracture toughness. With the addition of SiC, the maximum values were recorded at 3.2 MPa m^1/2^ at 3 wt% and 3.22 MPa m^1/2^ at 5 wt%, while the addition of 10 wt% TiB_2_ showed the highest fracture toughness value of 3.92 MPa m^1/2^. This increase is due to the roles that SiC and TiB_2_ play in inhibiting crack propagation and contributing significantly to the composite’s microstructure.

**Figure 6:**
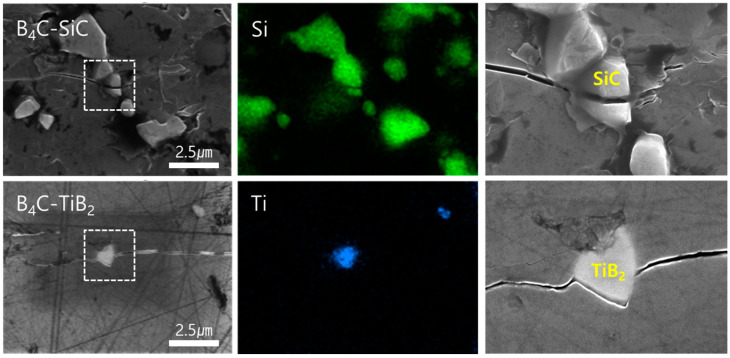
SEM images and EDS analysis of B_4_C-SiC/TiB_2_ composites, focusing on crack extension path; the images on the right side are enlarged images of the white box on the left.

The crack propagation mechanisms of SiC and TiB_2_ are different, but each plays a crucial role in improving the mechanical performance of the composite. SiC has a transgranular crack propagation mechanism in which cracks are blocked within the grains [3,4,10,11,23]. As shown in Figure 6, when SiC is evenly distributed, cracks are interrupted upon encountering SiC particles, slowing crack propagation and consuming more energy. This delay in crack propagation results from the strong interfacial bonding between SiC and B_4_C, attributed to the mutual solubility and diffusion-reaction at the interface during the hot-press sintering above 2000 °C. That is, the better interfacial compatibility of B_4_C and SiC phases increases the fracture toughness of the composite. Simultaneously, SiC inhibits grain growth, promoting the densification of the microstructure and enhancing the strength of the composite.

On the other hand, TiB_2_ primarily follows an intergranular fracture mechanism. Due to the difference in the coefficients of thermal expansion between TiB_2_ (8.1 × 10^−6^ K^−1^) and B_4_C (4.5 × 10^−6^ K^−1^), residual stress forms during the cooling process after sintering. This residual stress induces microcracks within the composite, propagating along the grain boundaries. The crack deflection results in a more complex propagation path, slowing the propagation speed and consuming more energy, thereby improving the fracture toughness [3,4,22,23,25]. Intergranular cracking caused by this residual stress is one of the critical mechanisms by which TiB_2_ enhances the fracture resistance of the composite. Even at 5 and 10 wt% of TiB_2_ additions, the uniformly distributed TiB_2_ particles contribute to maintaining and strengthening the mechanical performance of the composite through this mechanism.

As a result, SiC and TiB_2_ significantly improve the fracture toughness of B_4_C composites through their respective crack propagation and reinforcement mechanisms. SiC blocks cracks within the grains through its transgranular crack propagation mechanism and promotes microstructural densification by inhibiting grain growth. TiB_2_ complicates crack propagation, thereby inducing crack path deviation and residual stress at grain boundaries and significantly increasing the fracture resistance of the composite. These two mechanisms suggest that the mechanical performance of the composite can be optimized even with small amounts of additives (≤10 wt%). The enhanced crack resistance can also be attributed to the enhancement of flexural strength in the B_4_C composites, along with the reduced grain sizes that increase the density of grain boundaries to act as barriers for cracks. In particular, the combination of SiC and TiB_2_ plays a crucial role in simultaneously improving the performance of the composite, effectively enhancing the durability and mechanical properties of B_4_C-based composites.

## 4. Conclusions

In this study, B_4_C-SiC/TiB_2_ composites with small additions of SiC and TiB_2_ (<10 wt%) were prepared through hot-press sintering to not only maintain the light weight of B_4_C and its high level of hardness but also to enhance its strength and toughness. All composites achieved a relative density of over 95%, with only B_4_C, SiC, and TiB_2_ phases detected in the XRD patterns and no other impurity phases formed. Throughout the B_4_C matrix, even at the small additive amounts, the second-phase particles were uniformly distributed and inhibited grain growth. The minor additives also significantly impact the microstructure and mechanical properties of the composites. In the case of SiC, the grain size became uniform, and the strength and toughness increased, although the Vickers hardness decreased slightly. Meanwhile, by adding TiB_2_, the strength and toughness of the B_4_C were significantly improved without changing the Vickers hardness values despite the introduction of heavier TiB_2_ particles and a reduction in the relative density. Based on the reinforcement mechanisms derived from the mechanical properties and microstructures of the composites, we prove that even a minor amount of either SiC or TiB_2_ can play a crucial role as the secondary phase to enhance the performance of B_4_C itself, showing promise for the manufacturing of lightweight B_4_C-based materials.

## Figures and Tables

**Table 1:** Mechanical properties of B_4_C-based composites.

2nd Phase Content	Density (g/cm^3^)	% Relative Density	Elastic Modulus (GPa)	Vickers Hardness (GPa)	Fracture Toughness (MPa·m^1/2^)	Flexural Strength (MPa)
0 wt%	2.51	99.7	462	34.8 ± 0.2	2.56	515 ± 37
SiC 1 wt%	2.51	99.3	449	34.0 ± 0.1	3.04	548 ± 25
SiC 3 wt%	2.53	99.5	452	33.2 ± 0.6	3.2	540 ± 38
SiC 5 wt%	2.53	99.1	455	32.6 ± 0.2	3.22	551 ± 23
SiC 10 wt%	2.58	99.7	426	29.9 ± 0.4	3.1	605 ± 28
TiB2 5 wt%	2.56	97.9	446	34.8 ± 0.6	3.66	548 ± 27
TiB2 10 wt%	2.6	95.7	438	35.0 ± 0.4	3.92	544 ± 23

## Data Availability

The original contributions presented in the study are included in the article/Supplementary Material, further inquiries can be directed to the corresponding authors.

## Supplementary Materials

The following supporting information can be downloaded at: https://www.mdpi.com/article/10.3390/ma17246233/s1, Figure S1. Schematic illustration of the overall process steps for fabricating B_4_C-based composites, including powder mixing, drying, sieving, and hot pressing under vacuum. Figure S2. Microstructure of B_4_C-SiC composites with varying SiC contents (1%, 3%, 5% and 10%). Figure S3. High-magnification SEM images (BSE mode) of B_4_C-SiC composites. Figure S4. Microstructure and mechanical properties of B_4_C-TiB_2_ composites with varying TiB_2_ contents (5%, 10%); (a) flexural strength and Vickers hardness, (b) grain size of B_4_C, (c) SEM images. Table S1. Composition of Powder Mixtures. Table S2. Comparative Analysis of Sintering Methods for B_4_C-Based Ceramics.
