# Supplementary material for: Improving the Fracture Toughness of Boron Carbide via Minor Additions of SiC and TiB2 Through Hot-Press Sintering
Source: Materials (Basel). 2024 Dec 20;17(24):6233. doi: 10.3390/ma17246233 (PMC11678037; doi:10.3390/ma17246233)
Supplement: Supplementary file 1 [file materials-17-06233-s001.zip › materials-3309362-supplementary.pdf]

Supplementary Information

Improving the Fracture Toughness of Boron Carbide via Minor Additions of SiC and TiB_2 Through Hot-Press Sintering

Juhan Ka [1,2], Kyoung Hun Kim [1], Woohyuk Choi [3], Sungmo Jung [3,4], Tae Hwan Lee [3], Hyun Sik Kim [1], Heesoo Lee [2,*] and Jae Hwa Lee [1,*]

[1] Analysis & Standards Center, Korea Institute of Ceramic Engineering & Technology (KICET), 101 Soho-ro, Jinju-si 52851, Republic of Korea
[2] School of Materials Science & Engineering, Pusan National University, 2 Busandaehak-ro 63beon-gil, Busan 46241, Republic of Korea
[3] Material R&D Center, Samyang Comtech Co., Ltd., 81 Mansam-ro, Gwangju-si 12726, Republic of Korea
[4] Department of Materials Science & Engineering, Myongji University, 116 Myongji-ro, Yongin-si 17058, Republic of Korea
* Correspondence: heesoo@pusan.ac.kr (H.L.); jh.lee@kicet.re.kr (J.H.L.)

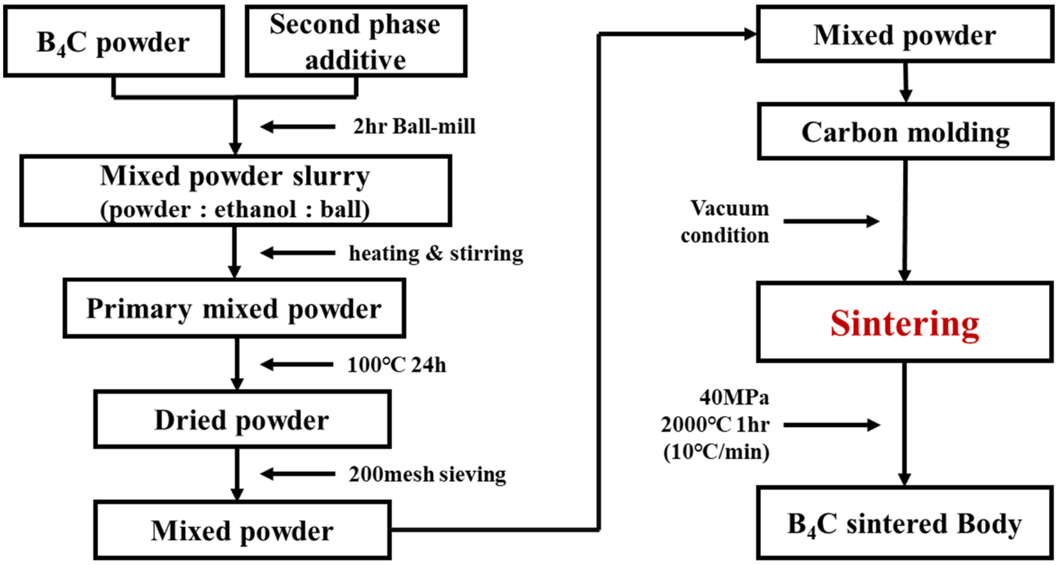

Figure S1. Schematic illustration of the overall process steps for fabricating B_4C-based composites, including powder mixing, drying, sieving, and hot pressing under vacuum.

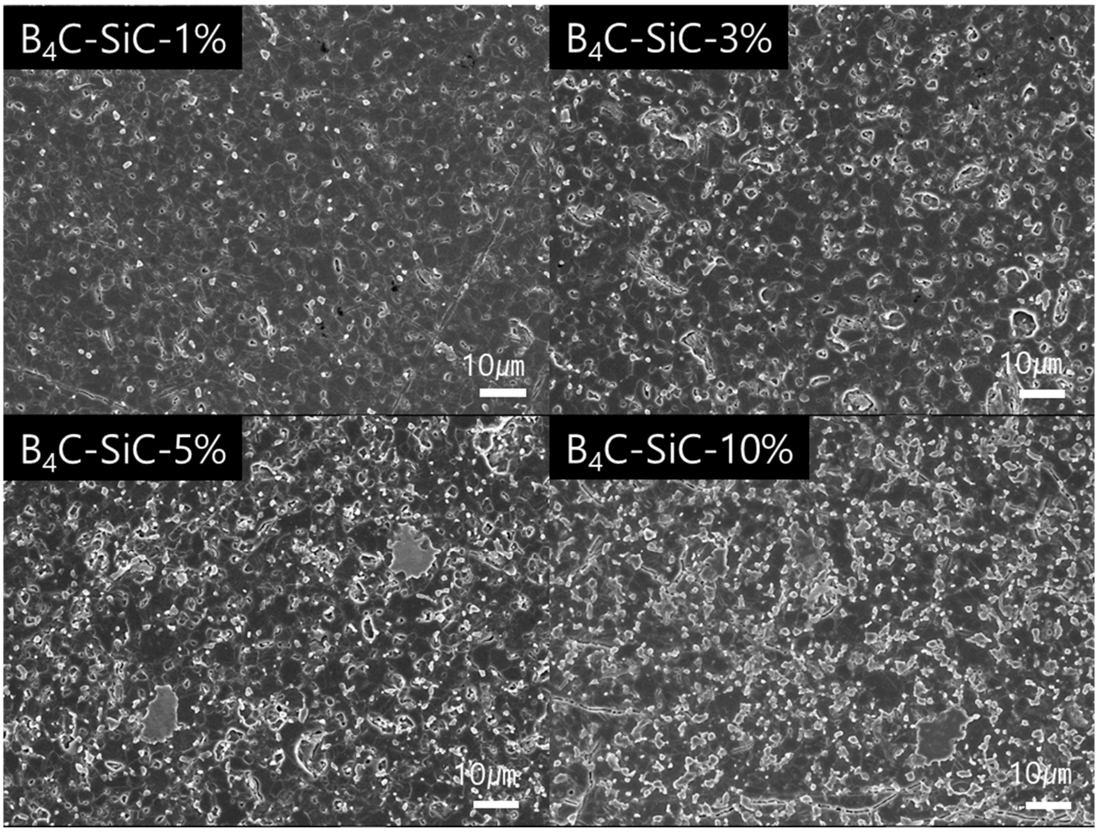

Figure S2. Microstructure of B_4C-SiC composites with varying SiC contents (1%, 3%, 5% and 10%).

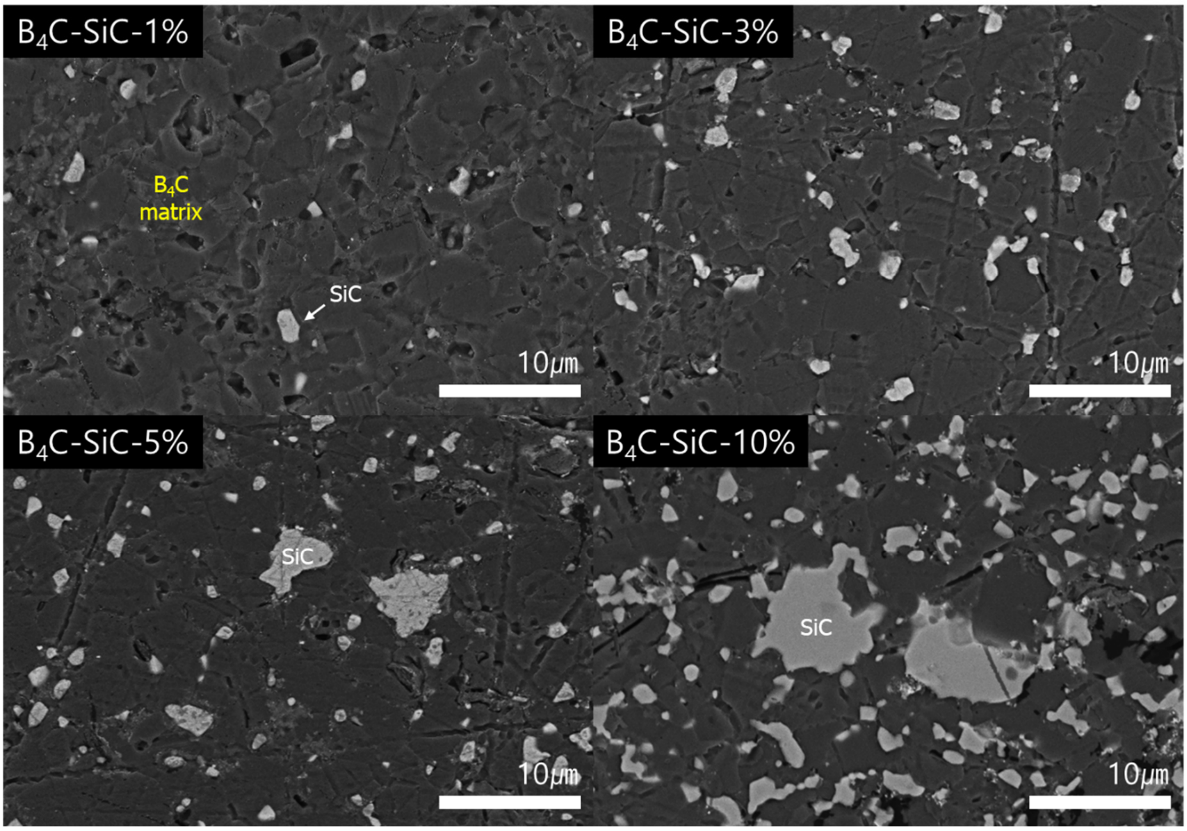

Figure S3. High-magnification SEM images (BSE mode) of B_4C-SiC composites

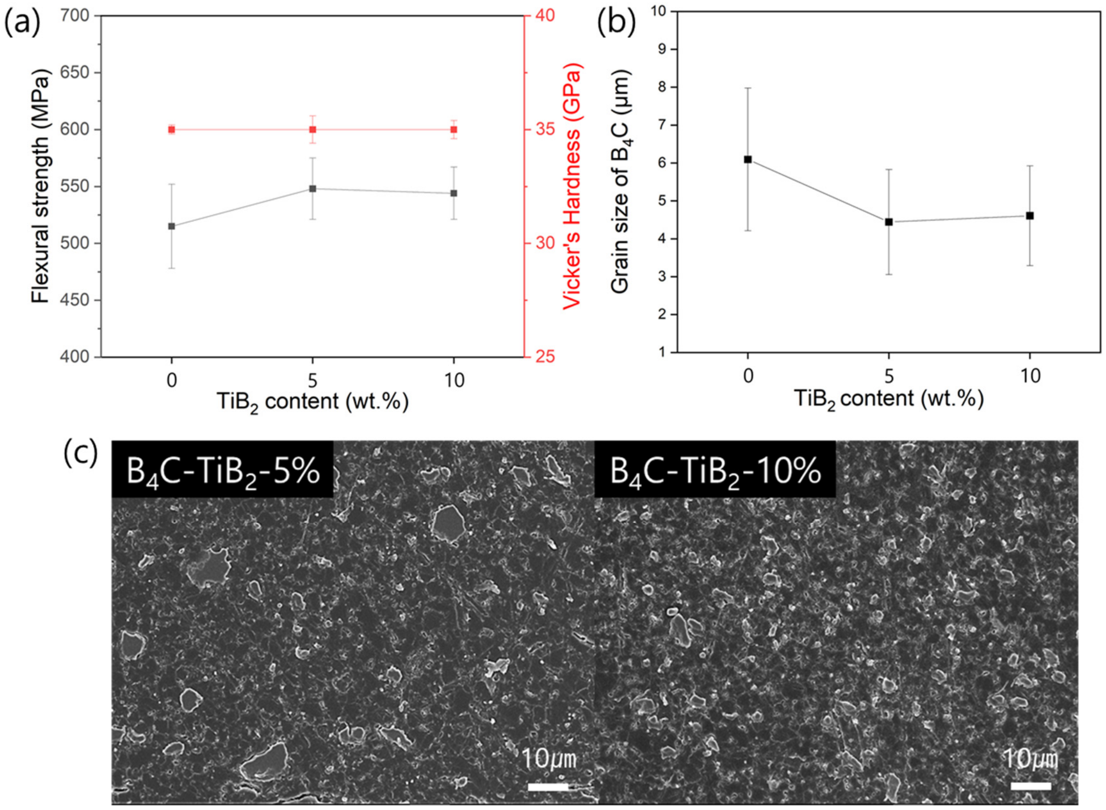

Figure S4. Microstructure and mechanical properties of B_4C-TiB_2 composites with varying TiB_2 contents (5%, 10%); (a) flexural strength and Vickers hardness, (b) grain size of B_4C, (c) SEM images

Table S1. Composition of Powder Mixtures

Materials	Composition
B_4C	100 wt.% B_4C
B_4C-SiC-1%	99 wt.% B_4C + 1 wt.% SiC
B_4C-SiC-3%	97 wt.% B_4C + 3 wt.% SiC
B_4C-SiC-5%	95 wt.% B_4C + 5 wt.% SiC
B_4C-SiC-10%	90 wt.% B_4C + 10 wt.% SiC
B_4C-TiB_2-5%	95 wt.% B_4C + 5 wt.% TiB_2
B_4C-TiB_2-10%	90 wt.% B_4C + 10 wt.% TiB_2

Table S2. Comparative Analysis of Sintering Methods for B_4C-Based Ceramics

Sintering Method	Sintering Temperature (°C)	Pressure (MPa)	Sintering Time (min)	Sample Size Capability	Key Advantages	Key Limitations
Hot Pressing (HP)	2000	~50	60	Large	Uniform microstructure, scalability, and reliable process	Requires high temperature and long processing time
Spark Plasma Sintering (SPS)	1850	~50	10	Small	Fast densification, energy-efficient process	Limited sample size, requires specialized equipment
Pressureless Sintering	>2100	-	>240	Large	Cost-effective, simple equipment setup	Low densification efficiency, long processing time
